# The Estimation of Economic Burden of Hepatitis C Virus Infection in Iran

**Published:** 2018-10

**Authors:** Mehdi MOHAMMADZADEH, Hamid DERAFSHI, Tayebeh GHARI

**Affiliations:** 1. Dept. of Pharmacoeconomy and Administrative Pharmacy, School of Pharmacy, Shahid Beheshti University of Medical Sciences, Tehran, Iran; 2. Pharmacoeconomy & Medical-Pharma Management Research Center, Tehran, Iran; 3. Dept. of Ophthalmology, School of Medicine, Alborz University of Medical Sciences, Karaj, Iran; 4. Dept. of Ophthalmology, School of Medicine, Iran University of Medical Sciences, Tehran, Iran; 5. Dept. of Pharmaceutics, School of Pharmacy, Alborz University of Medical Sciences, Karaj, Iran

**Keywords:** Hepatitis C, Economic burden of disease, Direct and indirect costs, DALYs, Iran

## Abstract

**Background:**

One of the major causes of liver-related mortality and morbidity is Hepatitis C Virus (HCV) infection. It is also one of the reasons behinds of chronic liver disease and related complications such as cirrhosis and hepatocellular carcinoma. This autoimmune liver disease imposes a high economic burden on individuals and the society. This study aimed to estimate burden of HCV in Iran.

**Methods:**

Overall, 200 patients with HCV infection, referred to hospitals in three cities of Tehran, Karaj and Tabriz, Iran during year 2015, were randomly enrolled. To estimate the total burden of hepatitis, direct and indirect costs, costs of DALYs and social welfare were calculated.

**Results:**

Economic burden of HCV infection was obtained 26242.8 purchasing power parity (PPP$). Intangible costs of HCV was calculated 207421.6 PPP$.

**Conclusion:**

Total direct costs of HCV for each patient are more than household consumption expenditure. Therefore, it is a reasonable policy to control and increase insurance coverage of HCV patients in order to decrease their costs.

## Introduction

One of the major causes of liver-related mortality and morbidity is Hepatitis C Virus (HCV) infection. It is also one of the reasons behinds of chronic liver disease and related complications such as cirrhosis and hepatocellular carcinoma (1, 2). Overall, 350,000 global deaths in a year have occurred because of HCV-related complications (3). HCV considered as an important disease in both developed and developing countries (4, 5). There are 160 million patients with HCV infection in the worldwide (6).

Transmission of this blood-borne pathogen will occur due to the exposure to the contaminated body fluids and blood (7, 8). Despite our knowledge about hepatitis C virology and pathogenesis has been grown rapidly, there is little information about the current and future burden of this infection throughout the world. It may potentially need considerable health care services and impose very high costs for health systems in the United States, Europe, and others. Therefore, it is essential to have the information about hepatitis burden in order to the development of national and international health policies for prevention of complications and control of disease and injury (9).

Several methods have been proposed to quantify population-level burden of disease. The disability-adjusted life years (DALYs) is one way to measure population health which is being increasingly used in comparing and analyzing the burden due to diseases (10). It is a concept similar to healthy year includes weights for time spent in less-than-perfect health. DALYs are formed from two components: years of life lost (LLY) because of premature death and years lived with disability (YLD) consist of nonfatal injuries and disease (11, 12). To measure the economic burden of hepatitis, a considerable number of studies have been published in the literature. The global epidemiology and burden of hepatitis C were measured. The total burden related to HCV may be as high as 4.2 million DALYs (8).

The average diagnosis and treatment costs of hepatitis C were studied. One course of treatment and six months after that with standard protocol plus Ribavirin and Peg-interferon plus Ribavirin have costs of exceeds 3,850 and 16,494 purchasing power parity dollars (PPP$) respectively (13). Hepatitis C is a very important and expensive disease for health care system. HCV patients need expensive drug treatments and require high health care services (13). A modeling approach was used to quantify the current HCV-infected population, future disease progression and associated costs in Egypt. With newer therapies, strategies to reduce disease burden are feasible and cost-effective (14). In one study, future morbidity, mortality, and costs resulting from HCV have been estimated. Number of deaths for chronic liver disease and hepatocellular carcinoma were estimated 165900 and 27200 deaths during year 2010 until year 2019 respectively. Indirect medical costs for HCV services were calculated $10.7 billion. From 2010 until 2019, HCV cause the loss of 1.83 million years of life in people younger than 65 with cost of $21.3 and $54.2 billion, respectively (15). In another study, cost-effectiveness of generic direct-acting antivirals (DAAs) for hepatitis C treatment was evaluated. By use of DAAs, the life expectancy increased by 8.02 years and the lifetime healthcare costs by $1,309 per-person treated in comparison of no treatment (16). To the best of our knowledge, the disability-adjusted life year’s estimation has not been done on HCV infection in Iran.

We aimed to estimate the population-level burden of HCV infection by calculating the disability-adjusted life years, direct and indirect costs and social welfare.

## Methods

Our study was descriptive, analytical, applied and retrospective. To do this study, 200 patients with HCV infection, who referred to hospitals in three cities of Tehran, Karaj and Tabriz, Iran during year 2015, were randomly selected.

Written informed consent was obtained from each participant. The study was approved by Ethics Committee of Shahid Beheshti University of Medical Sciences, Tehran, Iran.

Public library system and questionnaire were used for data collections. Questionnaire which contains information about demographic and patient’s history, quality of life, work productivity and activity impairment was prepared. Content validity of the questionnaire was examined with reference to the gastroenterologists’ and pharmacoeconomists’ comments and opinions. They were asked to evaluate relevance, completeness, scoring and clarity of each question. The Cronbach’s Alpha Coefficient for each question was determined to test the reliability of the questionnaire. Cronbach’s Alpha Coefficient more than 0.75 was approved level to each question. Correlation of each question with other questions was higher than medium level and there was no negative correlation (an inverse relationship) between questions.

After questionnaire preparation, a review of the literature was undertaken to recognize the latest treatments available for patients with HCV, in Iran. In order to calculate total economic burden of hepatitis C, the algebraic sum of direct medical costs, direct non-medical costs and indirect costs was obtained. Costs of DALYs and costs of economic welfare index were also calculated as intangible costs. These items were collected as following:

For direct medical and non-medical costs and indirect costs’ calculations, diagnostic and laboratory tests, physician visits and drug treatments were considered as direct medical costs and transportation, meals and telephone conversation were considered as direct non-medical costs. Common therapeutic protocol in HCV infection including direct-acting antivirals (DAAs) was used (6). We also calculated accommodation costs as direct non-medical costs. As most of patients were in their own cities and did not need accommodation. Therefore we eliminated accommodation costs from total non-medical direct costs. Days of absence from work (patient or his/her caregiver) were calculated as indirect costs by use of Laspeyres index.

The unit costs of these items (diagnostic and laboratory tests and physician visits) were calculated (http://irmna.com/). The prices of medication were retrieved from drug list of Food and Drug Organization of Iran (http://www.fda.gov.ir/en/).

To estimate the total burden of hepatitis, we also calculated DALYs (disability-adjusted life years) using Murray methodology (17). The years of life lost (YLL) because of premature mortality and the years lived with disability (YLD) are the two components of DALYs. Life expectancy was considered 74 and 77 year for men and women respectively based on life expectancy in Iran. Incidence, duration, and disability weight are the information specifically required for calculating YLD. For estimation of parts of economic burden based on calculated DALYs, we multiplied DALYs by GDP (gross domestic product) per capita. 15572.83 PPP$. PPP$ was used for the purpose of international comparisons. We also added economic welfare index for calculating the total economic burden of hepatitis. Welfare index was calculated by social welfare cardinal function as below:

$$W=\frac{1}{n}\sum_{i=1}^{n}Y_{i}=\bar{Y}$$

Where W is social welfare, Y_i_ is the income of individual and n is number of individuals. Finally, total economic burden of hepatitis C was obtained by the algebraic sum of direct medical costs, direct non-medical costs and indirect costs. Costs of DALYs and costs of economic welfare index were also calculated as intangible costs.

## Results

### Characterizations of patients with HCV infection

The study was done on 200 patients with HCV infection in Iran. The demographic characteristics of the mentioned patients have been shown in Table 1. Mean age (± standard deviation) of patients with HCV infection in our study was 35.5 ± 6.4. 165 patients (82.5 %) were male and majority of patients (132 (66%)) were married.

**Table 1:** Socio-economic characterizations of patients with HCV infection

***Variables***	***Frequency***	***Relative frequency (%)***
**Gender**		
Male	165	82.5
Female	35	17.5
**Age group(yr)**		
20–30	14	7
31–40	80	40
41–50	44	22
51–60	55	27.5
> 61	7	3.5
**Marital status**		
Single	68	34
Married	132	66

In Table 2, number of patients based on smoking, drinking, use of drugs and physical activities has been shown. Majority of patients (189 (94.5%), 147 (73.5%)) had no history of drinking and physical activities respectively; however, majority of patients (59.5%) had history of use of drugs.

**Table 2:** Smoking, drinking, use of drugs, physical activity, education and occupational status of HCV patients

***Variables***	***Frequency***	***Relative frequency (%)***
**Smoking**		
Yes	106	53
No	94	47
**Drinking**		
Yes	11	5.5
No	189	94.5
**Use of drugs**		
Yes	119	59.5
No	81	40.5
**Physical activities**		
Yes	53	26.5
No	147	73.5
**Occupation**		
Employed	137	68.5
Unemployed	63	31.5
**Education**		
Academic	53	26.5
Non academic	147	73.5

We have also evaluated HCV patients in terms of underlying diseases. Liver disease and dementia disease were the most prevalent underlying diseases with frequency of 120 (60 %) and 62 (31%) respectively. Education and occupational status of HCV patients have been displayed in Table 2. Most patients were employed but did not have an academic education.

### Direct medical and non-medical costs

For estimation of direct medical and non-medical costs of HCV infection, we calculated costs of laboratory and diagnostic tests, physician visits and drug treatments as direct medical costs and transportation, meals and telephone conversation as direct non-medical costs.

Common laboratory and diagnostic tests and their costs have been shown in Table 3. Total costs for tests was estimated about 900.9 $ equal to 2945.9 PPP$.

**Table 3:** Common laboratory and diagnostic tests and their costs for patients with HCV infection

***Laboratory and diagnostic test name***	***Unit price ($)***	***Frequency of test per year***	***Total costs per year ($)***
Viral load	89	2	178
PCR	138	4	552
Anti HCV	10	1	10
Liver biopsy	86.2	1	86.2
Liver sonography	11.5	1	11.5
ALT	2.3	4	9.2
AST	2.3	4	9.2
ALP	2.3	4	9.2
UA-UC	3.7	4	14.8
Bilirubin	2.9	4	11.6
Creatinine	2.3	4	9.2
Total costs			**900.9**

Associated costs for treatment have been presented in Table 4. Average drug treatment costs were 6368.3 $ equal to 20824.34 PPP$.

**Table 4:** Common therapeutic protocol in HCV infection and its associated costs for treatment

***Treatment***	***Unit price ($)***	***Number of drug per year***	***Annual costs ($)***
Treatment one			
Sofosbuvir Tablet	13.6	365	4964
Ribavirin Tablet	0.17	2190	372.3
PEG Interferon alfa 2a	43	48	2064
Total			**7400.3**
Treatment two			
Sofosbuvir Tablet	13.6	365	4964
Ribavirin Tablet	0.17	2190	372.3
Total			**5336.3**

Table 5 shows the annual direct costs of HCV. The total direct costs for HCV infection were 7399.9 $ equal to 24197.6 PPP$ annually. Minimum direct costs for HCV infection was costs of telephone conversation (47.1 PPP$) that it was 0.2% of total direct costs. The most expensive direct costs were also for drug treatment (20824.3 PPP$).

**Table 5:** Average annual direct medical and non-medical costs per patients with HCV infection

***Type of costs***	***Unit price ($)***	***Unit price PPP$***	***Percentage***
**Direct medical costs**			
Laboratory and diagnostic test	900.8	2945.6	12.2
Physician visits	34.5	112.8	0.5
Drug treatments	6368.3	20824.3	86
**Direct non-medical costs**			
Transportation	64.7	211.6	0.9
Meals	17.2	56.2	0.2
Telephone conversation	14.4	47.1	0.2
**Total**	**7399.9**	**24197.6**	**100**

### Indirect costs

Average indirect costs, ratio of direct to indirect costs and total costs of HCV infection were estimated in Table 6. Finally, total costs of direct and indirect costs of HCV infection were obtained 26242.8 PPP$.

**Table 6:** Average indirect costs, ratio of direct to indirect costs and total costs of HCV infection

***Type of costs***	***Unit price ($)***	***Unit price (PPP$)***
Indirect medical costs	625	2045.2
Ratio of direct to indirect costs	11.8	11.8
Total costs of direct and indirect costs	8025.3	26242.8

### DALYs (disability-adjusted life years)

DALYs are formed from years of life lost (LLY) because of premature death and years lived with disability (YLD) consist of nonfatal injuries and disease. In order to calculate the years of life lost (YLL) because of premature mortality, 150 patients passed away because of HCV were randomly selected and their age at death was determined. YLL was obtained 0.87 yr based on the differences between life expectancy and the average age at death in HCV patients.

Another dimension of the DALY is the time period in years lived in states of poor health or disability due to each disease (YLD). The disability is measured in length in years and in severity. The disability severity weights have been appointed by WHO for each disabling condition on a scale from one to zero. The mentioned scale, patients’ physical conditions, and experts’ opinions determine the weights. HCV patients’ physical conditions and reasons for their disability were evaluated. Reasons of patients’ disability were determined as insomnia, physical weakness, nervousness, depression, anxiety, joint and muscle pains, loss of appetite, inability to climb stairs, inability to flex knee, inability to walk more than 2 km and breathing problems. Average of severity weight for patients with HCV was obtained 0.34. HCV patients lived 15 yr in health states less than ideal health (with disability). YLD was calculated as follow:

YLD=Average of severity weight × years lived with disability=0.34×11=3.74

YLL and YLD were obtained about 0.8 and 3.74 yr respectively. Finally, DALYs formed from two components was obtained 4.6 yr. Costs of DALYs were gained from GDP per capita by means of YLL and YLD estimation. In Table 7, average costs of YLL, YLD, and DALY for each patient has been shown.

**Table 7:** Average costs of LLY and DLY of HCV infection

***Type of costs***	***Unit price ($)***	***Unit price (PPP$)***
Costs of YLL	4167.04	13626.2
Costs of YLD	17811.1	58242.4
Total costs of YLL and YLD (DALYs)	21938.2	71868.6

### Social Welfare index

A cardinal social welfare function is a function that takes as input numeric representations of individual utilities and returns as output a numeric representation of the collective welfare. Costs of social welfare were 135553.1 PPP$.

Finally, economic burden of HCV infection was gained by sum of direct costs and indirect costs. It was obtained 8025.3 $ equal to 26242.8 PPP$. Intangible costs of HCV were sum of costs of DALYs and costs of social welfare. It was calculated 63431.7 $ equal to 207421.6 PPP$.

## Discussion

HCV is one of the most common causes of morbidity, mortality and liver transplantation in the future. It also imposes a high socioeconomic burden on patients and the society (1, 18). Therefore, calculation of total costs of HCV is useful for healthcare systems to make a suitable health policy in the country. The aim of this study was to obtain total burden of HCV in Iran. Total direct medical and non-medical costs of HCV infection were estimated 24197.6 PPP$ annually per patient. Regarding the HCV patients population in Iran in 2015 (19), total direct costs for all HCV population in Iran have obtained 7,259,280,000 PPP$ annually from which the drug treatment costs had the largest share of it. These results are similar to the previous studies regarding high costs of drug treatments in HCV patients (20, 21). Although the prevalence of HCV infection in Iran is low, total direct costs of HCV population are dramatic which means that it has a high priority for our healthcare systems.

Some portions of direct medical costs of HCV will be decreased due to insurance coverage. According to the insurance coverage percentages, different behavior of the insurance organizations in Iran, share of paid by patients and portion of services covered by insurance, we estimate that up to 40% of direct medical costs will be paid by insurance and the remaining will be deducted from the household consumption expenditure. Therefore, about 4,393,356,000 PPP$ will be imposed on HCV patients population in Iran. As the total health costs in Iran in 2015 was 90,799,249,123 PPP$ (https://data.worldbank.org/), total direct costs of HCV population was equal to 4.8% of it. Since the household consumption expenditure per capita in 2015 was about 9962 PPP$ based on our results, total direct costs of HCV for each patient are more than the household consumption expenditure per capita. Therefore, for the HCV patient, household’s income is not enough to cover the costs of it. Another point is about indirect costs of HCV. It will impose 613,560,000 PPP$ on community and health care systems which is due to loss of productivity and can damage the economics of the society. Most of direct costs of HCV were spent on drug treatments. Therefore, it is a reasonable policy of our healthcare systems to increase insurance coverage of HCV drugs in order to decrease costs imposed on HCV patients.

One of the main measure indexes of disease frequency is incidence. As it is just based on patient’s number and basic population data, for comparing between different areas or different times, it must be standardized. Since DALYs consider both survival time and life quality, it is more inclusive than incidence (22). In addition, in the calculation of DALYs, standardization issues have been considered essentially. Therefore, it can be compared to areas or diseases directly (23, 24). Our results showed that the average DALYs was calculated 4.6 yr. According to the total HCV population in Iran, total DALYs for population was obtained 1,380,000 yr lost from which about 262,500 yr of it was years lost due to premature mortality. Equivalent to 262,500 yr will be deducted from life expectancy in the community.

## Conclusion

Total direct costs of HCV for each patient are more than household consumption expenditure and for the HCV patient, household’s income is not enough to cover the costs of this disease. Therefore, it is a reasonable policy of our health care systems to implement an effective control plan and increase insurance coverage of HCV drugs in order to decrease costs imposed on HCV patients or making a plan to have a more supportive program to cover their costs.

## Ethical considerations

Ethical issues (Including plagiarism, informed consent, misconduct, data fabrication and/or falsification, double publication and/or submission, redundancy, etc.) have been completely observed by the authors.
